# Cammidge Reaction

**Published:** 1913-04

**Authors:** 


Cammidge Reaction.—Libertini, *Riv. Crit. di Clin. Med.*, vtvb, No. 13, page 161. To test Schlumm and Hegler's statement that the C reaction was due to glucose, he checked the examinations by using both unfermented and fermented urines. In 50 persons a positive reaction was obtained in 37 before and in 33 after fermentation. A positive test was obtained in six out of 10 healthy persons and in 27 out of 36 diseases in which there was no clinical evidence of pancreatic lesion. He considers the test of no value.

